# Genetic variations in *ARE1* mediate grain yield by modulating nitrogen utilization in rice

**DOI:** 10.1038/s41467-017-02781-w

**Published:** 2018-02-21

**Authors:** Qing Wang, Jinqiang Nian, Xianzhi Xie, Hong Yu, Jian Zhang, Jiaoteng Bai, Guojun Dong, Jiang Hu, Bo Bai, Lichao Chen, Qingjun Xie, Jian Feng, Xiaolu Yang, Juli Peng, Fan Chen, Qian Qian, Jiayang Li, Jianru Zuo

**Affiliations:** 1 State Key Laboratory of Plant Genomics and National Plant Gene Research Center, Institute of Genetics and Developmental Biology, Chinese Academy of Sciences, 100101 Beijing, China; 2 University of Chinese Academy of Sciences, 100049 Beijing, China; 3 Shandong Rice Research Institute, Shandong Academy of Agricultural Sciences, 250100 Jinan, China; 4 State Key Laboratory of Rice Biology, China National Rice Research Institute, Chinese Academy of Agricultural Sciences, 310006 Hangzhou, China; 5 State Key Laboratory of Molecular Developmental Biology, Institute of Genetics and Developmental Biology, Chinese Academy of Sciences, 100101 Beijing, China; 6 Chinese Academy of Sciences Center for Excellence in Molecular Plant Sciences, 200032 Shanghai, China

## Abstract

In crops, nitrogen directly determines productivity and biomass. However, the improvement of nitrogen utilization efficiency (NUE) is still a major challenge in modern agriculture. Here, we report the characterization of *are1*, a genetic suppressor of a rice *fd-gogat* mutant defective in nitrogen assimilation. *ARE1* is a highly conserved gene, encoding a chloroplast-localized protein. Loss-of-function mutations in *ARE1* cause delayed senescence and result in 10–20% grain yield increases, hence enhance NUE under nitrogen-limiting conditions. Analysis of a panel of 2155 rice varieties reveals that 18% *indica* and 48% *aus* accessions carry small insertions in the *ARE1* promoter, which result in a reduction in *ARE1* expression and an increase in grain yield under nitrogen-limiting conditions. We propose that *ARE1* is a key mediator of NUE and represents a promising target for breeding high-yield cultivars under nitrogen-limiting condition.

## Abstract (web summary)

Understanding the regulatory mechanisms of nitrogen assimilation is crucial for developing crop cultivars with improved nitrogen utilization efficiency (NUE). Here the authors identify a new negative regulator of NUE and mutation of this gene increases 10–20% rice grain yield under nitrogen-limiting field conditions.

## Introduction

Nitrogen is an essential element for all living organisms and the nitrogen nutrients derived from plants are primary sources for humans and animals. In crops, nitrogen is one of the most predominantly limiting factors for productivity and breeding cultivars with the improved nitrogen utilization efficiency (NUE) is urgently demanding for sustainable development of agriculture^1,2^. Most non-legume plants absorb inorganic nitrogen compounds from soil, followed by converting inorganic into organic nitrogen compounds, a process known as nitrogen assimilation^2–5^. Plants mainly acquire nitrate (NO_3_^−^) and ammonium (NH_4_^+^) from soil by the plasma membrane-localized transporters. After entering the plant cell, nitrate is sequentially reduced to nitrite and ammonium by nitrate reductase and nitrite reductase, respectively, of which ammonium is converted into organic nitrogen compounds by the primary assimilation^2,4,6^.

The biochemical framework of nitrogen assimilation has been well established, while the underpinning regulatory mechanisms are not well understood^1,2,5^. The primary nitrogen assimilation is mediated by the coupled reactions catalyzed by glutamine synthetase (GS) and glutamate synthase, the latter one also known as glutamine:2-oxoglutarate amidotransferase (GOGAT). GS catalyzes the conversion of glutamate into glutamine by incorporating a molecule of ammonia, whereas GOGAT transfers an amide group from glutamine to 2-oxoglutarate to produce two molecules of glutamate. Two types of GOGAT, Fd-GOGAT and NADH-GOGAT, have been characterized, which use different reductants with distinctive tissue specificities and biochemical properties^7–9^. The GS/GOGAT cycle is highly conserved in the plant kingdom, ranging from algae to higher plants. In rice (*Oryza sativa* L.), weak mutant alleles of *fd-gogat* (also known as *abnormal cytokinin response1* or *abc1*) causes severe developmental defects and enhances bacterial blight resistance, whereas a null mutant allele is seedling lethal^10–12^. In this study, we functionally characterize a genetic suppressor of *abc1-1*, designated as *are1* (for *abc1-1 repressor1*), which partially rescues the nitrogen assimilation-deficiency phenotype of *abc1-1*. Null mutations in *ARE1* cause the delayed senescence, the enhanced NUE, and the increased grain yield under nitrogen-limiting conditions. We also find that the natural variations in the *ARE1* promoter are directly associated with its expression and grain yield, thereby identifying *ARE1* as a key mediator of NUE and a promising locus for the genetic improvement of NUE in rice.

## Results

### Characterization of the *are1* mutant

**Fig. 1:**
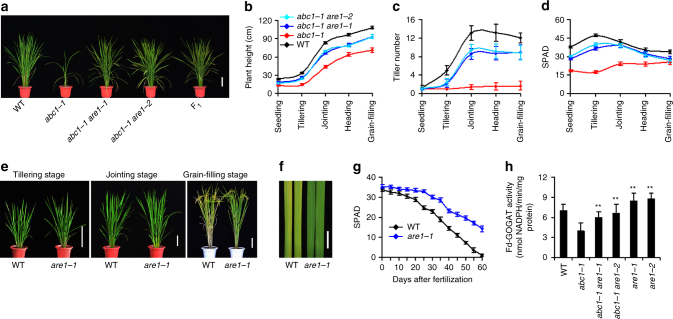
Identification and characterization of the *are1* mutants. **a** Plants at the heading stage with the indicated genotypes. F_1_ refers to F_1_ plants obtained from crosses between *abc1-1 are1-1* and *abc1-1 are1-2*. Scale bar, 15 cm. **b**–**d** Quantitative analysis of the plant height, tiller number, and soil-plant analysis development (SPAD, parameter indicating relative chlorophyll content) with the indicated genotypes at various growth stages. Data presented are mean values with s.d. (*n* = 30). **e** Wild-type (WT) and *are1-1* plants at the indicated growth stages. Scale bars, 15 cm. **f** Flag leaves of WT and *are1-1* plants 35 days post fertilization. Scale bar, 2 cm. **g** Quantitative analysis of SPAD in flag leaves of WT and *are1-1* plants at the indicated growth stages. Data presented are mean values with s.d. (*n* = 40). **h** Analysis of Fd-GOGAT activity in leaves derived from 3-week-old seedlings with the indicated genotypes. Data presented are mean values of 4 technical repeats with s.d. **P < 0.01 (Student’s *t*-test)

We previously identified a rice *abc1* mutant (in the Nipponbare or NPB background) that shows a typical nitrogen deficient syndrome, including the reduction in the plant height, tiller number, chlorophyll level, and grain yield^10^. *ABC1* encodes Fd-GOGAT, a key enzyme in the GS/GOGAT cycle for nitrogen assimilation^10^. We subsequently performed a genetic screen for *abc1-1 repressor* (*are*) mutants by examining the phenotype of the plant height, tiller number, and leaf color under field growth conditions. Two of those identified mutations, *are1-1* and *are1-2*, partially rescued the pleiotropic phenotype of *abc1-1*, including defects in the plant height, tiller number and the leaf chlorophyll content (Fig. 1a–d and Supplementary Fig. 1a). In an allelism test, all F_1_ progeny obtained from a cross between *are1-1* and *are1-2* showed an *abc1*-supressor phenotype (Fig. 1a and Supplementary Fig. 1a), demonstrating that these two mutations are allelic. The *abc1-1 are1-1* and *abc1-1 are1-2* double mutants were backcrossed with wild-type NPB plants three times, respectively, and the *are1-1* and *are1-2* single mutants were identified from respective BC_2_F_2_ progenies. These two alleles were phenotypically indistinguishable and *are1-1* was used in most experiments hereafter unless indicated otherwise.

During the early vegetative growth stages, *are1* showed a phenotype similar to wild type. At the later growth stages, *are1* had a slightly increased plant height and reduced tiller number (Fig. 1e). The heading date of *are1-1* was often delayed for 3–5 days than wild type. At the grain-filling stage, *are1-1* plants exhibited a stay-green phenotype (Fig. 1e, f). Consistent with this result, the accumulation of chlorophylls in *are1-1* was significantly higher than that in wild type after the heading stage (Fig. 1g), indicating that *are1-1* causes delayed senescence and prolonged efficient photosynthetic activities.

The *abc1-1* is a leaky mutant allele, retaining approximately 50–60% Fd-GOGAT activity, and the T-DNA insertional mutant *abc1-2* is a null allele^10^. Whereas the *are1-1* mutation did not cause detectable effects on the accumulation of Fd-GOGAT protein (Supplementary Fig. 1b), the Fd-GOGAT enzymatic activity in *abc1-1 are1-1* was nearly restored to that in the wild type (Fig. 1h). However, the *abc1-2 are1-1* double mutant showed an *abc1-2*-like or seedling-lethal phenotype (Supplementary Fig. 1c), indicating that *are1-1* is incapable of rescuing the phenotype of a null mutation in *ABC1/Fd-GOGAT*. These results suggest that the suppressing effect of *are1* on *abc1-1* is partly attributable to the rescued activity of Fd-GOGAT.

### Enhanced tolerance of *are1* to nitrogen deficiency

**Fig. 2:**
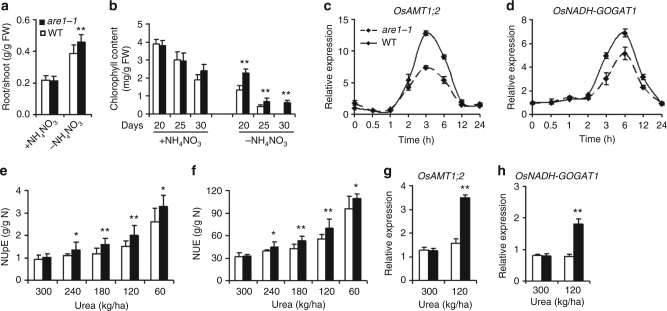
Enhanced tolerance of *are1-1* to nitrogen deficiency. **a** Analysis of the root/shoot ratio of 20-day-old wild-type (WT) and *are1-1* plants grown in the absence or the presence of NH_4_NO_3_ (1.46 mM). Data presented are mean values with s.d. (*n* = 40). **b** Quantification of chlorophyll content in wild-type and *are1-1* plants grown in the absence or the presence of NH_4_NO_3_ (1.46 mM) for the indicated times. Data presented are mean values with s.d. (*n* = 12). **c**, **d** Expression of *OsAMT1;2* and *OsNADH-GOGAT1* in response to nitrogen depletion. Two-week-old seedlings grown in a nitrogen-containing solution (see Methods) were transferred to a nitrogen-free solution (time 0), and then cultured for the indicated times. Total RNA was prepared from roots and used for qRT-PCR analysis. Data presented are mean values with s.d. (*n* = 12). **e**, **f** Analysis of nitrogen uptake efficiency (NUpE) and nitrogen utilization efficiency (NUE) of 5-month-old WT and *are1-1* plants grown under the indicated conditions. Data presented are mean values of 3 biological replicates with s.d. **g**, **h** Expression of *OsAMT1;2* and *OsNADH-GOGAT1* in 12-week-old WT and *are1-1* plants grown under the indicated conditions. Data presented are mean values of 3 technical replicates with s.d. *, **P < 0.05 and *P* < 0.01 (Student’s *t*-test), respectively

Given that the *are1* mutations largely rescue the nitrogen deficient phenotype of *abc1-1*, *ARE1* may be involved in nitrogen metabolism. Plants evolved various strategies for survival when grown under nutrient limiting environments, including the increase of the root-to-shoot ratio^13^. Compared to the wild type, *are1-1* showed an increased root-to-shoot ratio under nitrogen deficiency condition, mainly resulted from increased root biomass (Fig. 2a and Supplementary Fig. 2a, b). Consistently, *are1-1* seedlings retained a higher level of chlorophylls than the wild type (Fig. 2b and Supplementary Fig. 2c). Nitrogen depletion or reduction rapidly induced the expression of key genes involved in ammonia transport and nitrogen assimilation in wild-type rice seedlings, and this induction was reduced in *are1-1* (Fig. 2c, d and Supplementary Fig. 2d–k), indicating that *are1-1* is less sensitive to nitrogen starvation.

The reduced sensitivity of *are1* to nitrogen starvation may be contributed by the increased accumulation of nitrogen *in planta* when grown under sufficient nitrogen condition. To test this possibility, we analyzed the nitrogen content in the field-grown plants. The total nitrogen contents in various organs were significantly higher in *are1-1* than that in wild type plants, and the nitrogen uptake efficiency (NUpE) and NUE were consequently increased when the nitrogen fertilizer was progressively reduced (Fig. 2e, f and Supplementary Table 1). Consequently, the expression of the ammonia transport and nitrogen assimilation genes was increased in *are1-1* than that in wild type plants under limited nitrogen supplies (Fig. 2g, h and Supplementary Fig. 2l–n), suggesting that *ARE1* is negatively correlated to NUpE and NUE.

### Molecular characterization of *ARE1*

**Fig. 3:**
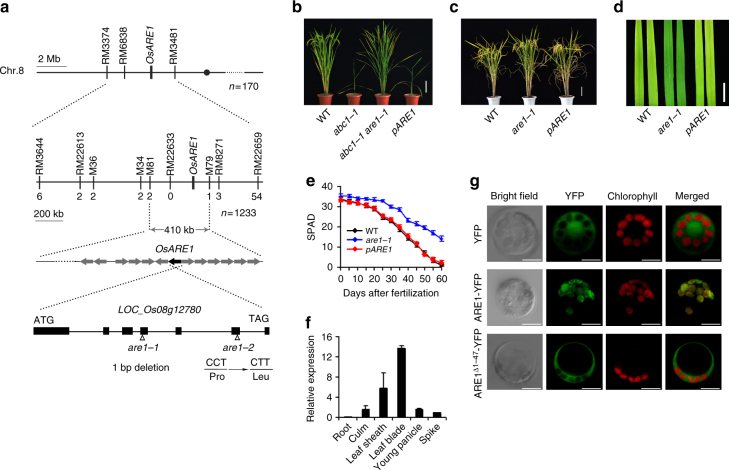
Molecular characterization of *ARE1*. **a** Genetic mapping of *ARE1*. The numbers (*n*) of recombinants used in mapping are given below the genetic maps (see Methods for details). Bottom, a schematic illustration of the *ARE1* structure. Filled boxes and lines represent exons and introns, respectively. The mutation natures of *are1-1* and *are1-2* are shown below. **b**, **c** Molecular complementation of the *are1-1* mutant phenotype. Plants at the grain-filling and dough stages with the indicated genotypes are shown. *pARE1*: *abc1-1 are1-1* (**b**) and *are1-1* (**c**) plants carrying a pARE1::ARE1 transgene, respectively. **d** Flag leaves derived from plants shown in (**c**). **e** Analysis of soil-plant analysis development (SPAD) in flag leaves of plants with the indicated genotypes at various growth stages. Data presented are mean values with s.d. (*n* = 40). **f** Analysis of the *ARE1* expression in various organs by qRT-PCR. Data presented are mean values with s.d. (*n* = 3). **g** Analysis of subcellular localization of ARE1-YFP (yellow fluorescent protein) protein in rice protoplasts. Scale bars, 15 cm in (**b**, **c**), 2 cm in (**d**), and 5 μm in (**g**)

In a genetic analysis, all F_1_ progeny (18 plants) obtained from a cross between *are1-1* and NPB showed a wild-type-like phenotype by analyzing the senescence phenotype. In the F_2_ population, the normal and delayed senescence phenotypes were segregated approximately in a ratio of 3:1 (561:196 = 2.86:1; *χ*^2^_C_ < *χ*^2^_0.05_ = 3.84), indicating that *are1* is a recessive Mendelian mutation in a single nuclear gene. Using a map-based cloning approach, we genetically mapped *ARE1* to chromosome 8 in a region containing 13 predicted open reading frames (ORFs) (Fig. 3a; see Methods for details). DNA sequencing analysis revealed the presence of a single base deletion (A-899; the putative transcription start is referred to as +1) in the fourth exon of *LOC_Os08g12780* in the *are1-1* genome, resulting in frame-shift mutations after this base (Fig. 3a and Supplementary Fig. 3). In the *are1-2* genome, a single base substitution (C-T) in the sixth exon caused a highly conserved Pro residue being substituted with Leu (Fig. 3a and Supplementary Fig. 3).

To verify the identity of the *ARE1* candidate gene, we performed a molecular complementation test by transforming of *abc1-1 are1-1* plants with a wild-type genomic fragment containing the entire *LOC_Os08g12780* gene under the control of its native promoter (~2.3 kb) and 3′-untranscribed region (~1.9 kb). All the T_1_ transgenic plants showed an *abc1-1*-like phenotype (Fig. 3b), indicating that the transgene reverts the suppressing effect of *are1-1* on the *abc1-1* mutation. When transformed into *are1-1* plants, the transgene rescued the delayed senescence phenotype of the mutant (Fig. 3c–e). These results demonstrate that *LOC_Os08g12780* represents *ARE1*.

*ARE1* encodes a putative protein that is highly conserved from lower to higher plants with unknown function (Supplementary Figs. 3 and 4a). Overall, ARE1 shares approximately 27–85% identity with ARE1-like proteins from cyanobacteria to flowering plants (Supplementary Fig. 4a). In the rice genome, a putative protein encoded by *LOC_Os02g24598* shares low homology with ARE1 (22.6% identity). *ARE1* is predominately expressed in photosynthetic tissues, including leaves and leaf sheaths, with a lower expression level in culms, young panicles and spikes (Fig. 3f and Supplementary Fig. 4b–d). ARE1 was predicted as a chloroplast-localized protein containing a putative transit signal peptide of 47 amino acid residues (Supplementary Figs. 3 and 4e, f). When transiently expressed in rice protoplasts, ARE1-YFP (yellow fluorescence protein) fusion protein was localized in chloroplasts, whereas the deletion of the putative transit signal peptide retained the fusion protein in the cytoplasm (Fig. 3g).

### Increased grain yield of *are1* under low nitrogen conditions

Data presented above indicate that *are1* is an important mediator of NUE. To assess the potential contribution of *ARE1* to grain yield, we performed a multi-year field trial to analyze the major agronomic traits of *are1-1* plants grown under various nitrogen conditions, in comparison with the wild-type NPB variety. The experiment was repeated for four years (2013–2016) and each sample included at least three duplicates. Under our assay condition, the saturated nitrogen fertilizer concentrations are approximately 280–300 kg/ha urea, similar to the routine usage by farmers in most rice-growing areas in China^14^.

**Fig. 4:**
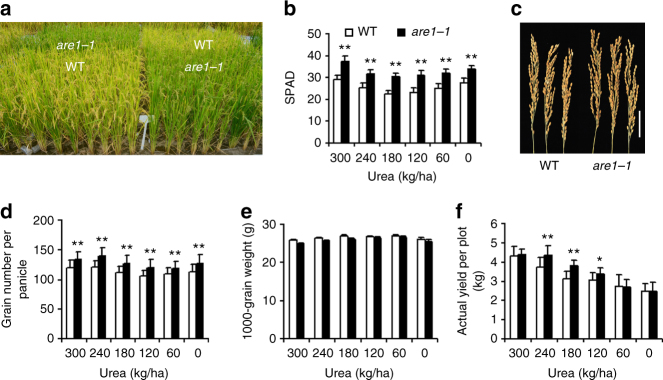
Enhanced grain yield by *are1*. **a** Grain-filling stage wild-type (WT; the *japonica* Nipponbare variety) and *are1-1* plants grown under 180 kg/ha urea. See Supplementary Fig. 5a for plants grown under other nitrogen supply conditions. **b** Analysis of soil-plant analysis development (SPAD) in flag leaves of WT and *are1-1* plants shown in (**a**). Data presented are mean values of 5 biological replicates with s.d. **c** Panicles of WT and *are1-1* plants. Scale bar, 5 cm. **d**–**f** Analysis of grain number per panicle, 1000-grain weight, and grain yield per plot of WT and *are1-1* plants grown under various urea concentrations as indicated (*n* = 40 in (**d**, **e**), and *n* = 12 in (**f**)). Error bars in (**b**, **d**–**f**) indicate s.d. *, **P < 0.05 and *P* < 0.01 (Student’s *t*-test), respectively

During early growth stages, no substantial difference was observed between NPB and *are1-1* plants. However, after heading, *are1-1* showed significantly delayed senescence than NPB under various nitrogen conditions (Fig. 4a and Supplementary Fig. 5a), accompanying with increased photosynthetic activities (Fig. 4b) and higher nitrogen utilization efficiency (Fig. 2e, f and Supplementary Table 1). Compared to that of NPB, although the plant height and the tiller number of *are1-1* were marginally altered, the panicle length and the numbers of primary and secondary branches of panicles were increased in *are1-1*, resulting in an increase in grain number per panicle (Fig. 4c, d and Supplementary Fig. 5b–f). However, one-thousand-grain weight of *are1-1* was slightly reduced than that of NPB (Fig. 4e). Overall, the grain yield of *are1-1* plants increased by 10–20% than NPB grown under the nitrogen-limiting condition (40–60% of the saturated concentration; Fig. 4f and Supplementary Fig. 5g, h), under which conditions the differences of NUpE and NUE between NPB and *are1-1* were also most apparent (Fig. 2e, f and Supplementary Table 1). Most notably, the grain yield of *are1-1* grown under the nitrogen-limiting condition was comparable to that of NPB grown under the saturated urea condition (Fig. 4f and Supplementary Fig. 5g, h). These results indicate that *are1-1* is a beneficial allele of high NUE and is particularly useful under nitrogen-limiting growth conditions.

### Analysis of genetic variations in *ARE1*

**Fig. 5:**
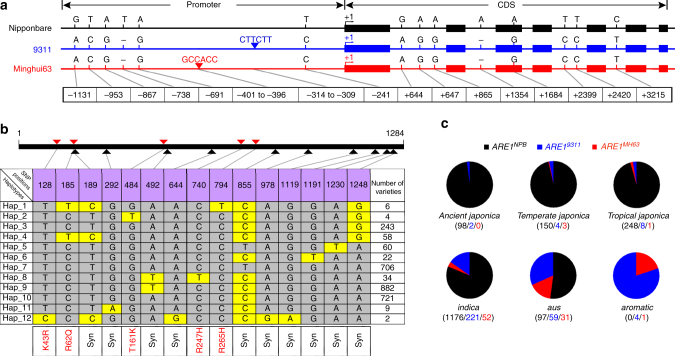
Analysis of genetic variations in *ARE1*. **a** Schematic representation of genetic variations in *ARE1* in a *japonica* variety Nipponbare (NPB) and two *indica* varieties 9311 and Minghui63 (MH63). Exons are shown by filled boxes and other sequences are shown by lines. Numbers at the bottom indicate positions of variations (the putative transcription start is referred to as +1). CDS, coding sequences. **b** Major haplotypes of single nucleotide polymorphisms (SNPs) in the *ARE1* coding region. Major SNP haplotypes and casual variations in the encoded amino acid residues are shown. The *ARE1* coding sequences of 2747 rice varieties were compared with that of NPB (Hap_7). Twelve haplotypes were identified from these accessions and polymorphic nucleotides of each haplotype are highlighted by yellow boxes. The numbers of the identified varieties of each haplotype are shown at right. Syn, synonymous variations. **c** Distribution of three haplotypes of insertion-deletion polymorphisms (InDels) in the *ARE1* promoter in various accessions. The numbers of the detected haplotypes (specified by different colors) are given below each group

Given that *ARE1* plays an important role in mediating NUE and grain yield, we then explored potential association between these traits and genetic variations in *ARE1*. By analyzing a panel of Asian cultivated rice accessions from the 3000 Rice Genomes Project^15,16^, multiple variations were identified in the *ARE1* promoters and coding sequences (Fig. 5a, b). In a panel of 2747 accessions, 15 single nucleotide polymorphisms (SNPs) were identified in the coding region, which were classified as 12 major haplotypes (Fig. 5b). Among these 15 SNPs, five variations caused substitutions in the coded amino acid residues, of which all occurred at very low frequencies (0.07–2.33%; Fig. 5b). The functional significance of these five SNPs remains elusive. In 2155 accessions with available data in the promoter region, three major haplotypes, *ARE1*^*NPB*^, *ARE1*^*9311*^, and *ARE1*^*MH63*^, were identified, represented by the NPB, 9311 and Minghui63 (MH63) promoters, respectively. In these three haplotypes, three insertion–deletion polymorphisms (InDels) were identified in the promoter region of *ARE1*, of which the *ARE1*^*9311*^ and *ARE1*^*MH63*^ haplotypes contained two small insertions of 6-bp at different positions (Fig. 5a).

Because of relatively more dramatic alterations in the genome structure, we focused on the analysis of the InDel polymorphisms in the *ARE1* promoter. We found that the majority of *japonica* varieties were the *ARE1*^*NPB*^ haplotype, with only 2.00, 3.50, and 4.46% occurrence of the *ARE1*^*9311*^/*ARE1*^*MH63*^ alleles in the ancient *japonica*, *temperate japonica*, and *tropical japonica* accessions, respectively (Fig. 5c). However, the occurrence of the *ARE1*^*9311*^/*ARE1*^*MH63*^ alleles was increased to 18.84% in the examined 1449 *indica* accessions, and higher frequency of the *ARE1*^*9311*^/*ARE1*^*MH63*^ alleles was observed in *aus* (48.13%) and *aromatic* (100%) (Fig. 5c), suggesting that the *ARE1* locus has been likely subjected to artificial selection during breeding.

### Genetic variations modulate *ARE1* expression and grain yield

**Fig. 6:**
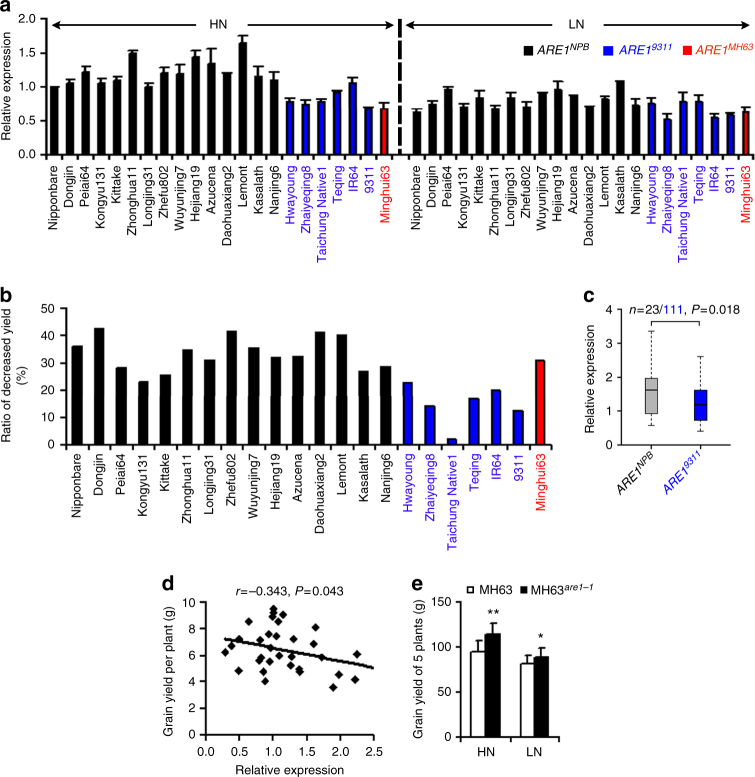
Modulation of *ARE1* expression and grain yield by InDel polymorphisms. **a** Analysis of the *ARE1* expression in representative varieties with three different InDel haplotypes. Total RNA prepared from leaves of 3-week-old seedlings hydroponically grown under high nitrogen (HN; 1.46 mM NH_4_NO_3_) or low nitrogen (LN; 0.73 mM NH_4_NO_3_) conditions was used for qRT-PCR analysis. Data presented are mean values of three technical replicates with s.d. **b** The ratio (HN vs. LN) of decreased grain yield per plants shown in (**a**). **c** Analysis of the *ARE1* expression in the Liangyoupei9 (LYP9) recombinant inbred lines (RILs; F_13_ generation). The numbers (*n*) of each haplotype and *P* value (Student’s *t*-test) are given above the graph. Total RNA was prepared as defined in (**a**) and data presented are mean values of three technical replicates with s.d. **d** Analysis of the correlation of grain yield with the *ARE1* expression level in RILs shown in (**c**). *r* and *P* values are determined by the Pearson correlation analysis. Leaves of plants at the heading stage grown under low nitrogen conditions (150 kg/ha urea) were used for the preparation of total RNA and subsequent qRT-PCR analysis. Data presented are mean values of three technical replicates with s.d. **e** Gain yield of MH63 and MH63 near isogenic lines carrying an *are1-1* allele (MH63^*are1-1*^; BC_6_F_4_) grown under high nitrogen (HN; 240 kg/ha) or low nitrogen (LN; 120 kg/ha) conditions. Data presented are mean values with s.d. (*n* = 20 plants). *, **P < 0.05 and *P* < 0.01 (Student’s *t*-test), respectively

To evaluate the biological function of these InDels, we examined the *ARE1* expression levels in representative *indica* and *japonica* accessions of different haplotypes. All the analyzed *ARE1*^*9311*^/*ARE1*^*MH63*^ haplotypes, regardless of their genetic backgrounds, had a decreased expression level of *ARE1* than the *ARE1*^*NPB*^ haplotypes under high nitrogen growth condition (Fig. 6a). Moreover, whereas low nitrogen treatment repressed the *ARE1* expression, the *ARE1*^*NPB*^ haplotypes were more sensitive to low nitrogen than the *ARE1*^*9311*^/*ARE1*^*MH63*^ haplotypes (Fig. 6a). This result suggests that the *ARE1* expression is negatively regulated by nitrogen availability and the genetic variations in the promoter of the *ARE1*^*9311*^/*ARE1*^*MH63*^ accessions cause the reduced promoter activity and the responsiveness to nitrogen. Correlated to these results, the differences of grain yield between high and low nitrogen growth conditions in accessions carrying an *ARE1*^*NPB*^ allele were generally greater than that of those carrying an *ARE1*^*9311*^/*ARE1*^*MH63*^ allele (Fig. 6b and Supplementary Fig. 6a), suggesting that the two insertions in the *ARE1*^*9311*^ and *ARE1*^*MH63*^ haplotypes are negatively correlated to both the *ARE1* expression level and grain yield.

To further test whether these InDels are functional in a similar genetic background, we analyzed the phenotypes of a set of recombinant inbred lines (RILs) generated from crosses between two *indica* rice varieties 9311 and Peiai64 (PA64), also known as Liangyoupei9 (LYP9), a widely cultivated high-yield hybrid rice^17^. Of these two parents, PA64 was characterized as an *ARE1*^*NPB*^ haplotype. In a panel of 134 RILs of LYP9, the *ARE1*^*9311*^ InDel was tightly co-segregated with the phenotype of the reduced *ARE1* expression level (Fig. 6c), which, in turn, was negatively correlated to the grain yield under nitrogen-limiting conditions (Fig. 6d and Supplementary Fig. 6b).

In a transgenic study, overexpression of *ARE1* caused the reduction in grain yield (Supplementary Fig. 6c, e, f). Conversely, the knockdown of the *ARE1* expression by RNAi caused an increased grain yield under nitrogen-limited conditions (Supplementary Fig. 6d, g, h), indicating that the expression level of *ARE1* is directly and negatively correlated to the grain yield.

*Indica* rice varieties generally have higher NUE than *japonica* varieties^18–20^. We next asked if the *are1-1* allele, which is in the *japonica* variety NPB genetic background, was also advantageous for high-yield *indica* varieties. In a near-isogenic line of MH63 carrying an *are1-1* allele (MH63^*are1-1*^), the photosynthetic activity was substantially higher than its parent MH63, accompanying with an increase in the grain number and, eventually, an increase in grain yield (Fig. 6e and Supplementary Fig. 6i, j). Taken together, these results suggest that the genetic variations in the *ARE1* promoter directly regulate its expression, thereby the rice productivity under nitrogen-limiting conditions.

## Discussion

In this study, we have presented multiple lines of evidence demonstrating that *ARE1* is a key determinant of the grain yield by modulating NUE in rice. Loss-of-function mutations in *ARE1* largely rescue the nitrogen deficient syndrome caused by impaired nitrogen assimilation associated with the reduced Fd-GOGAT/ABC1 activity^10,11^, indicating that *ARE1* is a negative regulator of nitrogen assimilation. ARE1 is a chloroplast-localized protein with unknown function. Along with its subcellular localization, the enhanced photosynthetic activity and delayed-senescence phenotype of *are1* mutants imply that ARE1 is not only involved in nitrogen assimilation, but may also be related to carbon metabolism. Consistent with this notion, the homologous gene of *ARE1* in algae is implied in regulating photosynthesis by modulating CO_2_ transport^21–23^. ARE1 shares approximately 27% homology with cotA protein of *Synechocystis* PCC6803 (see Supplementary Fig. 4a), which is also localized in chloroplasts. Mutations in the *cotA* gene caused reduced CO_2_ transport activity, presumably resulting from defects in light-induced proton extrusion^21,22^. In *Chlamydomonas reinhardtii*, the chloroplast-localized Ycf10 protein, which shares homology with cotA, has been shown to promote inorganic carbon (CO_2_ and HCO_3_^-^) uptake into chloroplasts^23^. Although the molecular mechanism of cotA-mediated and Ycf10-mediated inorganic carbon transport remains elusive, a similar biochemical activity may also be utilized by ARE1-like proteins in higher plants to modulate photosynthesis, which provide the carbon skeletons, energy and reductants for nitrogen metabolism. Understanding the precise biochemical function of ARE1 will be crucial to reveal the mechanism of ARE1-mediated carbon and nitrogen metabolism. Because the GS/Fd-GOGAT cycle is a key converging point of C/N balance that is directly linked to the TCA cycle^24,25^, a key question to be addressed is the biochemical and molecular mechanisms of the repressing effect of ARE1 on the Fd-GOGAT activity.

Genetically, a suppressor may act upstream or downstream of the modified locus. Because *are1-1* partially rescued the phenotype of the weak allele *abc1-1*, but not that of the null allele *abc1-2*, the *ARE1* function should be dependent on *ABC1*. Therefore, *ARE1* may genetically act upstream of *Fd-GOGAT/ABC1*. In agreement with this notion, whereas *gogat1*, which carries a substitution mutation in *Fd-GOGAT/ABC1*, shows an early senescence phenotype and an increase in grain nitrogen deposition^11^, *are1-1* shows an opposite phenotype, further suggesting that *ABC1* and *ARE1* act in a linear genetic pathway. Moreover, we notice that the *ARE1* expression level is substantially increased in the rice *grain number, plant height, and heading date7* (*ghd7*) mutant^26,27^, indicating that the expression of *ARE1* is negatively regulated by *Ghd7*. As implied from its name, *Ghd7* regulates multiple traits, including the grain number and chlorophyll contents^26–28^, which are also affected by the *are1* mutations. We speculated that *Ghd7* may act genetically upstream to regulate the *ARE1* expression, thereby modulating a subset of similar physiological and developmental processes.

The structure of the *ARE1* promoter shows significant variations among a large pool of rice varieties. The two small insertions in the *ARE1* promoter are rarely found in all *japonica* varieties, but the occurrence is significantly increased in *indica* and *aus* accessions, implying that these genetic variations may have been artificially selected during breeding. We speculate that these two InDels were selected in some *indica* and *aus* varieties under certain circumstances, likely using the grain number per panicle as a selectable trait. This notion may explain the high frequency of these two InDels in *aus*, a class of varieties that are widely grown in the south Asia areas where the nitrogen deposition in soil is significantly lowered than other regions^29–31^. Finally, when grown under nitrogen-limiting conditions, the grain yield of both *japonica* and *indica* varieties carrying an *are1-1* allele is comparable to that of their parents supplied with the saturated amount of nitrogen fertilizers. Therefore, discoveries made in this study present a potential solution of reducing the application of nitrogen fertilizers in rice production. Because of the conservation of *ARE1*-like genes, a similar approach may also be applicable to other crops.

## Methods

### Plant materials and growth conditions

The *abc1-1* mutant is a weak mutant allele carrying a single substitution mutation in *Fd-GOGAT*/*ABC1* in the Nipponbare background, and the *abc1-2* mutant is a T-DNA insertion mutant (PFG_3A-01082) in the Dongjin background^10^. The *abc1-1* seeds were mutagenized by ethyl methanesulfonate and M_2_ seeds (approximately 28800 seeds) obtained from self-pollinated M_1_ plants were used for screening of putative suppressors under field growth conditions. The screen was performed by mainly analyzing of the plant height, tiller numbers and leaf color during the primary round of screen. Putative *are* mutants were subjected to the secondary round of screen in the M_3_ generation and then backcrossed with wild-type (Nipponbare; NPB) or *abc1-1* mutant plants for at least three times. The MH63^*are1*^ plants were constructed by backcrossing of *are1-1* with MH63 for seven times, and resulting BC_6_F_4_ population were used in this study. The LYP9 recombinant inbred lines (RILs) were generated from a cross between an *indica* variety 9311 and a maternal *indica* variety PA64, a photo-thermosensitive male sterile line^17^. The inbred F_13_ generation was used in this study.

Plants were grown in paddy fields in Beijing, Jinan (Shandong province), Hangzhou (Zhejiang province), and Lingshui (Hainan province) with routine management. For hydroponic grown seedlings, seeds were surface-sterilized with 75% ethanol for 5 min and then sterilized with 30% bleach for 30 min, followed by washing with sterile water for six times. Sterilized seeds were germinated in distilled water for 36–48 h at 37 °C in darkness, and then transferred to a modified hydroponic culture solution^32^ and fresh solution was changed every 3 days. The modified hydroponic culture solution contains variable concentrations of NH_4_NO_3_ (0, 0.15, 0.73, or 1.46 mM), 0.37 mM CaCl_2_, 0.55 mM MgSO_4_, 0.18 mM KH_2_PO_4_, 0.09 mM K_2_SO_4_, 46.26 μM H_3_BO_3_, 0.32 μM CuSO_4_, 0.77 μM ZnSO_4_, 9.15 μM MnCl_2_, 0.38 μM K_2_MoO_4_, 20 μM Fe-EDTA, and 0.36 mM Na_2_SiO_3_, pH5.5. When transferring between hydroponic culture solutions containing different concentrations of NH_4_NO_3_, seedlings were washed in distill water for 3–5 times. Rice seedlings were cultured in a greenhouse or a growth chamber with 70% relative humidity under 12 h/12 h light/dark and 25–30 °C/25 °C day/night conditions.

Genetic transformation of rice was performed as described^33,34^. Brifly, rice embryonic calli were infected with agrobacteria cells for 1–2 min, followed by extensive washing with sterile water. The infected calli were cultured on N6-AS medium (3.981 g/L N6 salts, 2 mg/L 2,4-D, 0.3 g/L casein hydrolysate, 30 g/L sucrose, 10 g/L glucose, 15 mg/L acetosyringone, 4 g/L phytagel, pH 5.2) for 3 days at 25 ^o^C and then washed with sterile water containing 500 mg/L carbenicillin for three times. The calli were cultured on selection medium (3.981 g/L N6 salts, 2 mg/L 2,4-D, 0.5 g/L casein hydrolysate, 0.5 g/L l-proline, 30 g/L sucrose, 4 g/L phytagel, 200 mg/L carbenicillin, 200 mg/L cefotaxime, 50 mg/L hygromycin B, pH 5.8) for 4 weeks by changing fresh selection medium once. Putative positive transformants (hygromycin B-resistant calli) were then transferred to differentiation medium (4.4 g/L MS salts, 30 g/L sucrose, 30 g/L sorbitol, 2 g/L casein hydrolysate, 0.02 mg/L NAA, 2 mg/L kinetin, 4 g/L phytagel, 50 mg/L carbenicillin, 50 mg/L hygromycin B, pH 5.8) and cultured for 4–8 weeks. The regenerated plantlets were cultured on rooting medium (2.2 g/L MS salts, 0.5 g/L L-proline, 0.5 g/L casein hydrolysate, 30 g/L sucrose, 0.5 mg/L IBA, 4 g/L phytagel, pH 5.8) for several weeks and then grown in soil in a greenhouse upon rooting.

### Field trials

A randomized block design approach was used to analyze the major agronomic traits under various nitrogen conditions. Rice plants were cultivated with a distance of 15 × 25 cm in a 3.8–4.0 × 4.0 m plot and each sample or treatment included six replicates (plots). Urea was used as the only nitrogen source and applied for three times at the seedling, tillering and booting stages, with 30, 40, and 30% of total applied urea at each stage, respectively. Potassium sulfate (120 kg/ha) and calcium phosphate (260 kg/ha) were used as phosphorus and potassium fertilizers, supplied when rice seedling transplanting.

### Plasmid construction

Plasmids were constructed following standard methods as described^35^. To construct pARE1::ARE1, three partially overlapped DNA fragments (5000, 2000, and 3227 bp, respectively) were PCR-amplified using NPB genomic DNA as templates and primers embedded with appropriate restriction sites. The PCR fragments were first cloned into a pBluescript SK (Stratagene) vector and then sequentially inserted into the *Pst*I/*Sac*I sites of a binary vector pCAMBIA1300.

To construct p35S::ARE1-FLAG plasmid, a 279-bp NOS terminator fragment was PCR-amplified and then inserted into the *Sac*I/*Eco*RI sites of pCAMBIA1300 to generate a pCAMBIA1300-NOS vector. A 1295-bp fragment was PCR-amplified using NPB cDNA as a template and cloned into the *Pst*I/*Xma*I sites of pBluescript SK, in-frame fused to a FLAG tag to generate an *ARE1-FLAG* fusion gene, which was then released by *Pst*I/*Xba*I and inserted into the *Pst*I/*Xba*I sites of pCAMBIA1300-NOS to generate pARE1-FLAG-NOS. A 35S promoter fragment was PCR-amplified and then inserted into the *Pst*I site of pARE1-FLAG-NOS to generate p35S::ARE1-FLAG.

To construct pUbi::RNAi-ARE1, a 324-bp fragment (nucleotides 101–400; the first nucleotide of the putative translation codon was referred to as +1) was PCR-amplified using NPB cDNA as a template and then inserted into the pTCK303 vector in an inverted orientation^36^.

To construct p35S::ARE1-YFP, a 1296-bp fragment was PCR-amplified using NPB cDNA as a template and then inserted into *Hin*dIII/*Sal*I sites of pSAT6-EYFP-N1 vector^37^. The p35S::ARE1^Δ1-47^-YFP vector was constructed using a similar approach.

To construct pARE1::GUS, the *GUS* gene was inserted into *Bam*HI/*Sac*I sites of pCAMBIA1300-NOS vector to generate pCAMBIA1300-GUS-NOS. A 2.3-kb DNA fragment spanning the putative *ARE1* promoter region (−2276 to +6; the putative transcription start is referred to as +1) was PCR-amplified using NPB genomic DNA as a template. The PCR fragment was digested with *Pst*I (embedded in the PCR primers) and then inserted into the *Pst*I site of pCAMBIA1300-GUS-NOS vector.

All constructs were verified by DNA sequencing analysis and extensive restriction digestion. Sequences of all primers used in cloning are listed in Supplementary Table 2. All binary vector-based plasmids were transformed into *Agrobacterium tumefaciens* strain EHA105 by electroporation and the resulting bacteria cultures were used for the transformation of rice.

### Map-based cloning

F_2_ mapping populations were generated by crossing *abc1-1 are1-1* (in the *japonica* NPB background) with *indica* varieties MH63 and Nanjing6, respectively. F_2_ segregants showing rescued leaf chlorosis of *abc1-1* were selected for genetic mapping. Using 170 recombinants, the *ARE1* locus was tentatively mapped to an interval between two SSR markers, RM3374, and RM3481, on the short arm of chromosome 8. Using additional 1233 recombinants, *ARE1* was mapped to a 410-kb region between two newly developed SNP markers M79 and M81. The *ARE1* candidate gene was identified by DNA sequencing analysis. Sequences of all primers used in genetic mapping are listed in Supplementary Table 2.

### Reverse transcription-quantitative PCR analysis

Total RNA from various organs was extracted using RNAprep Pure Plant Kit (TIANGEN, Beijing, China). Total RNA (~2 μg) was treated with DNase I for 10 min at room temperature, and then used for cDNA synthesis using TransScript^®^ First-Strand cDNA Synthesis SuperMix (TransGen Biotech, Beijing, China) according to the manufacturer’s instructions. Quantitative PCR experiments were performed with gene-specific primers using UltraSYBR Mixture (CWBIO, Beijing, China) in a CFX96^TM^ Real-Time System (BIO-RAD, US). The rice *Ubiquitin* (*UBQ*) gene was used as an internal control for normalization. Sequences of all primers used in qPCR are listed in Supplementary Table 2.

### Analysis of GUS activity

The GUS (β-glucuronidase) reporter activity was assayed by histochemical staining as described^38^. Briefly, Various tissues/organs derived from pARE1::GUS transgenic plants at the seedling stage (seedling), the jointing-booting stage (leaf sheath, leaf blade, and panicle) or the heading stage (spike) were collected and incubated in 90% acetone for 15 min at 4 °C. After washing three times with GUS staining solution [10 mM EDTA, 78.4 mM Na_2_HPO_4_, 31.6 mM NaH_2_PO_4_, 0.5 mM K_4_Fe(CN)_6_, 0.5 mM K_3_Fe(CN)_6_, 0.1% Triton X-100, and 20 mM 5-bromo-4-chloro-3-indolyl-β-D-glucuronide (X-Gluc)] and evacuation, samples were incubated in the staining solution for 2–24 h at 37 °C in darkness. The samples were destained by dehydration with 70% ethanol and then photographed.

### Immunoblotting analysis

The preparation of protein extracts and immunoblotting analysis were performed as described previously^10,34^. Leaves were grinded into fine powder in liquid nitrogen and total proteins were extracted in cold extraction buffer (50 mM HEPES, pH 7.5, 15 mM KCl, 1 mM EDTA, 1 mM DTT, and 1 mM PMSF) at 4 °C. The sample was cleaned by centrifugation at 20000×*g* at 4 °C for 10 min twice and the supernatant was collected. The sample was mixed with equal volume of 2× loading buffer (100 mM Tris–Cl, pH 6.8, 1.0% β-mercaptoethanol, 0.2% bromophenol blue, 20% glycerol, 4% sodium dodecyl sulfate), boiled for 1–2 min, and then subjected to SDS–PAGE. After gel electrophoresis, the proteins were electrically transferred onto a polyvinylidene difluoride membrane and analyzed by immunoblotting. A rabbit anti-Fd-GOGAT antibody (Agrisera; Cat #: AS07242; 1:20000 dilution) and a mouse anti-HSP82 antibody (Beijing Protein Innovation; Cat #: AbM51099-31-PU; 1:10000 dilution) were used as primary antibodies and HPR-conjugated goat anti-rabbit IgG or HPR-conjugated goat anti-mouse IgG (Dingguo Changsheng Biotech, Beijing, China; 1:50000 dilution) were used as secondary antibodies. The signal was detected using a SuperSignal West Femto Maximun Sensitivity Substrate kit (Thermo Scientific, Cat #: 34096) according to the manufacturer’s instructions.

### Transient expression assays in rice protoplasts

Rice protoplasts were prepared as described with minor modification^39^. Briefly, stem and sheath tissues derived from 14-day-old rice seedlings were cut into 0.5–1.0 mm strips. The sample was immediately transferred into 150 mL of 0.6 M mannitol and incubated for 10 min, followed by incubation in an enzyme solution (0.6 M mannitol, 10 mM MES, pH 5.7, 10 mM CaCl_2_, 5 nM β-mercaptoethanol, 1.5% cellulase RS, 0.75% macerozyme R-10, 0.1% BSA, and 50 μg/mL carbenicillin) for ~4 h at 28 °C with gentle shaking (40–60 rpm) in darkness. Protoplasts were collected using a 35 μm mesh filter and resuspended in one volume of W5 solution (154 mM NaCl, 125 mM CaCl_2_, 5 mM KCl, and 2 mM MES, pH 5.7) by inverting for 5–8 times, followed by centrifugation at 150×*g* for 3 min. Protoplasts were then resuspended in MMG solution (0.6 M mannitol, 15 mM MgCl_2_, and 4 mM MES, pH 5.7) at a concentration of 1–5 × 10^6^ cells/mL. The viability of protoplasts was determined by the FDA staining method as described^40^.

For transient expression assays, 5 μg plasmid DNA were introduced into 50 μL freshly prepared protoplasts by PEG-mediated transformation as described^41^. Briefly, 55 μL freshly prepared PEG solution (0.6 M mannitol, 100 mM CaCl_2_ and 40% PEG4000) was gently added, and then incubated for 15 min at 28 °C in darkness, followed by gently mixed with ten volumes of W5 solution and inverted for 5–8 times. The protoplasts were pelleted by centrifugation at 150×*g* for 3 min, and then gently resuspended in W5 solution.

For the analysis of subcellular localization, after transformation with appropriate expression vectors, protoplasts were cultured for 12–16 h in darkness at 28 °C, and then fluorescence signals were visualized and scanned under a confocal laser scanning microscope (Leica TCS SP5). At least 50 cells were analyzed in each sample.

### Analysis of Fd-GOGAT enzyme activity

Analysis of Fd-GOGAT enzyme activity was performed as described^10,42^ with minor modifications. Briefly, plant extracts were prepared by grinding 2 g fresh leaves of 3-week-old seedlings in 1 mL of cold extraction buffer (50 mM HEPES, pH 7.5, 15 mM KCl, 1 mM EDTA, 1 mM DTT, and 1 mM PMSF) at 4 °C, and then cleared by centrifugation at 20000×*g* for 10 min at 4 °C, followed by collection of the supernatants on ice. To assay Fd-GOGAT activity, 200 μL plant extracts (~240 μg protein) were mixed with 800 μL reaction mixture [50 mM HEPES, pH 8.5, 1% (v/v) β-mercaptoethanol, 3.65 mM glutamine, 3 mM α-oxoglutarate, 0.2 mM NADPH, and 0 or 4 μM ferredoxin], and then transferred into a quartz cuvette incubated in a DU^®^ 800 Nucleic Acid/Protein Analyzer (Beckman Coulter, US) for the Kinetics/Time Run. The Fd-GOGAT activity [nmoles NADPH oxidized (37 °C, pH 8.5)/min/mg protein] was measured spectrophotometrically by recording the rate of NADPH oxidation at 340 nm (indicated by a change in A_340_ nm blanked using reaction mixture without the addition of NADPH), and corrected by the rate measured from the reaction mixture without ferredoxin. All experiments were repeated at least three times (biological replicates), and mean values of these replicates are presented.

### Determination of total chlorophyll content and SPAD

To measure total chlorophyll contents, 10–15 mg leaves was collected in 2 mL 96% ethanol, and then homogenized in a MM 400 grinding apparatus (Retsch, Germany), followed by incubation at 4 °C for 2 h. The chlorophyll content was calculated spectrophotometrically based on the absorbance of the supernatant at 649 and 665 nm as described^43^. The relative chlorophyll content in leaves of plants grown in the paddy field was determined by a soil plant analysis development (SPAD) value measured by an SPAD-502 Plus chlorophyll meter (Konica Minolta, Japan) following the manufacturer’s instructions.

### Determination of total nitrogen content

To measure total nitrogen contents, samples were incubated in a drying oven at 105 °C for 30 min, followed by incubation at 90 °C for 5 days. Dry weights were measured as biomass values, and total nitrogen of various organs was determined by the Kjeldahl method as described^32,44^. The nitrogen uptake efficiency (NUpE) was determined by the ratio of total aboveground plant nitrogen to applied fertilizer nitrogen, and the nitrogen use efficiency (NUE) was determined by the ratio of total grain yield to applied fertilizer nitrogen, respectively, as described^45^.

### Bioinformatics analysis

Prediction of the subcellular localization and cleavage site of ARE1 protein were used TargetP 1.1 Server (http://www.cbs.dtu.dk/services/TargetP/) with cutoff value 0.500 according to the online instructions^46^. Prediction of transmembrane helices in ARE1 protein was performed via TMHMM 2.0 Server (http://www.cbs.dtu.dk/services/TMHMM/). Construction of the phylogenetic tree was performed using MEGA 5.10 software by Neighbor-Jointing method with 1000 bootstrapping trails.

### Data availability

The authors declare that all data supporting the findings of this study are available within the manuscript or its supplementary files or are available from the corresponding authors upon request.

## Electronic supplementary material

Supplementary Information
