# Supplementary material for: Genetic variations in ARE1 mediate grain yield by modulating nitrogen utilization in rice
Source: Nat Commun. 2018 Feb 21;9:735. doi: 10.1038/s41467-017-02781-w (PMC5821702; doi:10.1038/s41467-017-02781-w)
Supplement: Supplementary file 1 — Supplementary Information [file 41467_2017_2781_MOESM1_ESM.doc]

**Genetic variations in *ARE1* mediate grain yield by modulating nitrogen utilization in rice**

Wang et al.


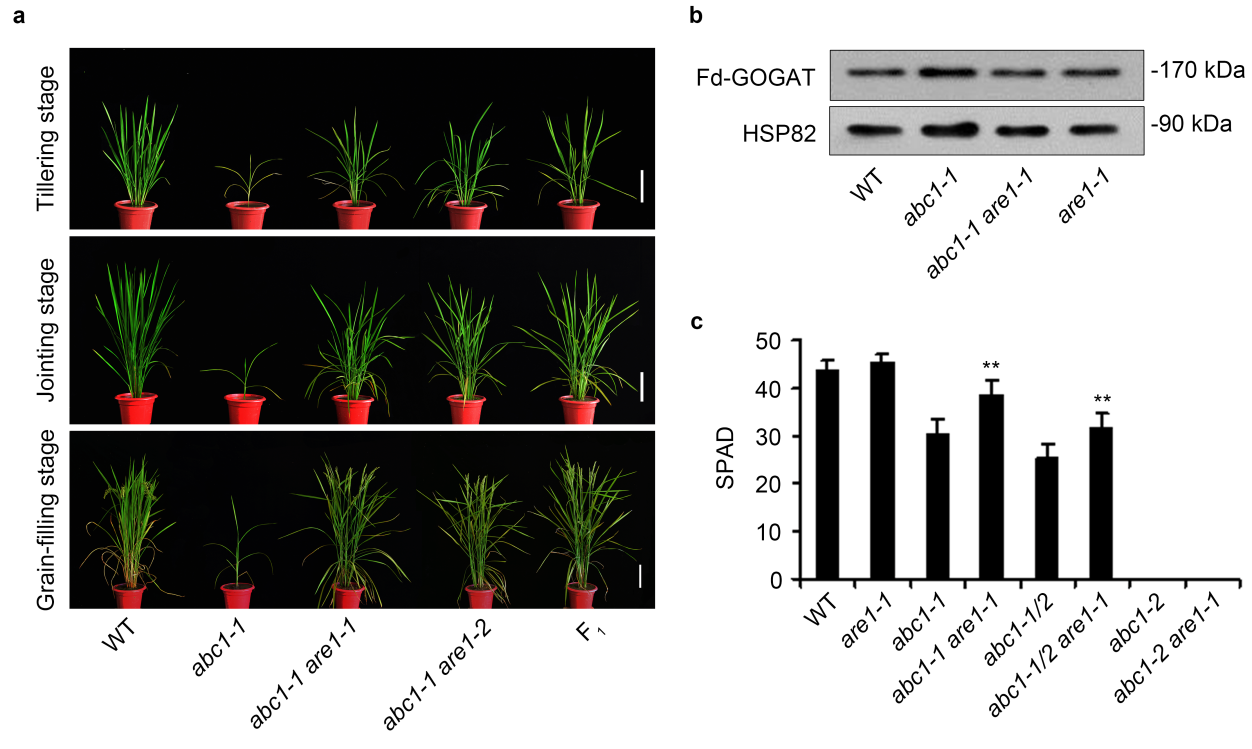


**Supplementary Figure 1 Identification and characterization of the *are1* mutant.** (**a**) Wild-type (WT) and various mutant plants at various growth stages with the indicated genotypes. F_1_ refers to F_1_ plants obtained from crosses between *abc1-1 are1-1* and *abc1-1 are1-2*. Scale bars, 15 cm. (**b**) Analysis of Fd-GOGAT/ABC1 protein levels in leaves of 3-week-old seedlings by immunoblotting. The rice HSP82 protein was used as a loading control. (**c**) Analysis of soil-plant analysis development (SPAD) of top leaves derived from the tillering stage plants with the indicated genotypes. *abc1-1/2* refers to the *abc1-1/abc1-2* heterozygous plants. Data presented are mean values of 3 technical replicates with s.d. ** indicates *P* < 0.01 (Student’s *t*-test).


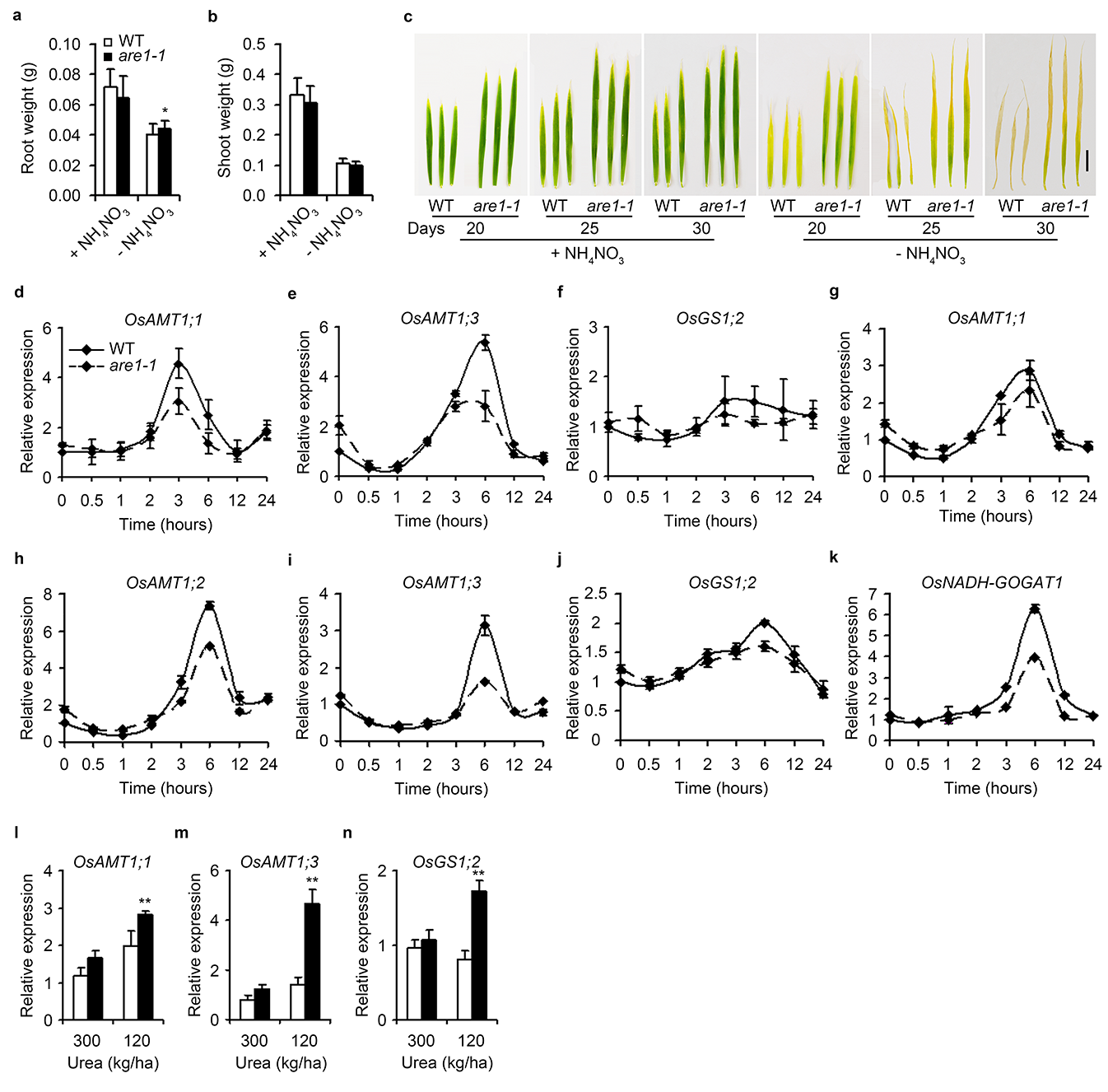


**Supplementary Figure 2 Enhanced tolerance of *are1-1* to nitrogen deficiency.** (**a**,**b**) Quantitative analysis of root and shoot weight of 20-day-old wild-type (WT) and *are1-1* plants grown in the absence or the presence of NH_4_NO_3_ (1.46 mM). Data presented are mean values of 3 technical replicates with s.d. (**c**) The first leaf blades derived from wild-type (WT) and *are1-1* plants grown in the absence or the presence of NH_4_NO_3_ (1.46 mM) for the indicated times. Scale bar, 1 cm. (**d**-**f**) Expression of *OsAMT1;1*, *OsAMT1;3*, and *OsGS1;2* in response to nitrogen depletion. Data presented are mean values of 3 technical replicates with s.d. See **Figure 2c**,**d** for technical details. (**g**-**k**) Expression of *OsAMT1;1*, *OsAMT1;2*, *OsAMT1;3*, *OsGS1;2*, and *OsNADH-GOGAT1* in response to nitrogen reduction (0.15 mM NH_4_NO_3_). Data presented are mean values of 3 technical replicates with s.d. (**l**-**n**) Expression of *OsAMT1;1*, *OsAMT1;3*, and *OsGS1;2* in 12-week-old WT and *are1-1* plants grown under the indicated conditions. Data presented are mean values of 3 technical replicates with s.d. * and ** indicate *P* < 0.05 and *P* < 0.01 (Student’s *t*-test), respectively.


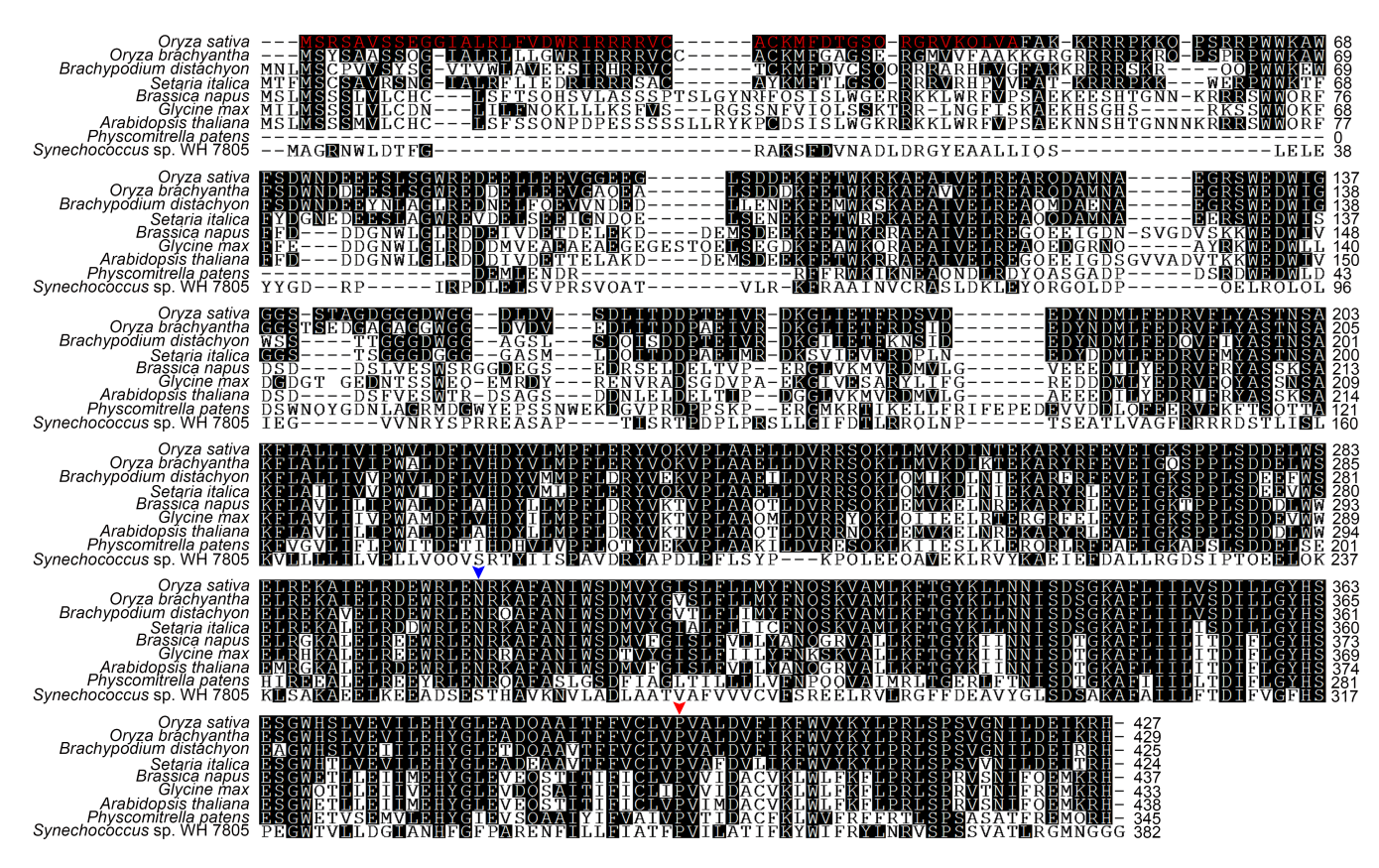


**Supplementary Figure 3 Sequence alignment of ARE1-like proteins.** Alignment of ARE1 and ARE1-like proteins using MegAlign software by the Clustal W method. The putative transit signal peptide sequences are highlighted in red. The mutated residues in *are1-1* and *are1-2* are highlighted by blue and red arrow heads, respectively. Accession numbers for the aligned sequences: *Oryza sativa*: XP_015648443; *Oryza brachyantha*: XP_015695716; *Brachypodium distachyon*: XP_014757144; *Setaria italica*: XP_012702526; *Brassica napus*: XP_013678677; *Glycine max*: XP_003516797; *Arabidopsis thaliana*: NP_567865; *Physcomitrella patens*: XP_001771702; *Synechococcus* sp. WH 7805: WP_006041495.

**
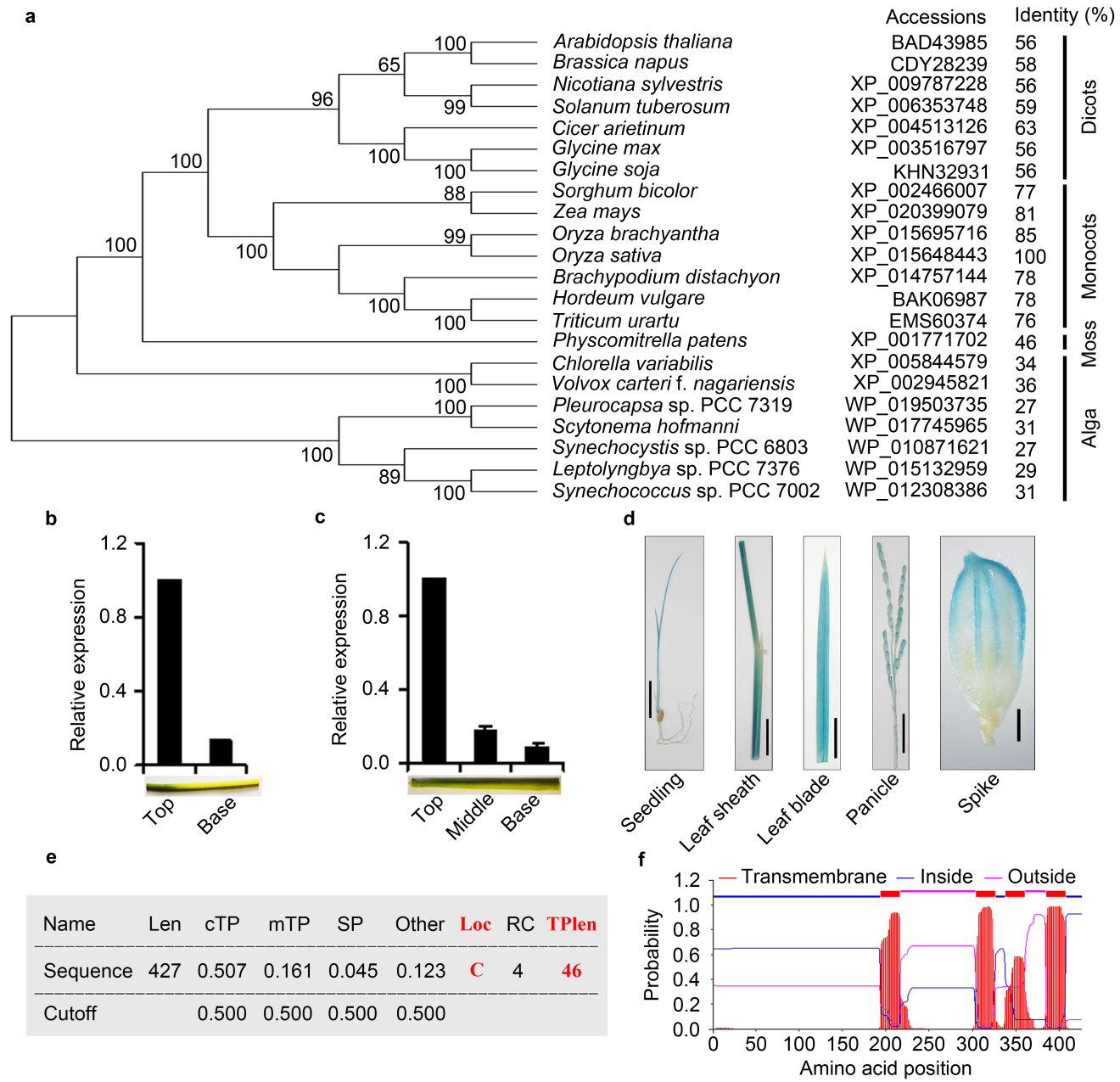
**

**Supplementary Figure 4 Molecular analysis of ARE1.** (**a**) Phylogenetic analysis of ARE1 and ARE1-like proteins in various species. The phylogenetic tree was constructed using MEGA 5.10 software by Neighbor-Jointing method with 1000 bootstrapping trials. The numerals on the branch sites indicate bootstrapping values. Bootstrapping values over 50% are shown in the phylogenetic tree. The accession numbers and the identity with ARE1 of ARE1-like proteins are shown at the right. (**b**,**c**) The relative expression levels of *ARE1* in different parts of culm (**b**) and leaf sheath (**c**). Data represent mean values with s.d. (*n* = 3). (**d**) Analysis of the p*ARE1*::*GUS* expression in seedlings and the indicated organs/tissues. Three independent transgenic lines were analyzed and similar results were obtained. Scale bars, 1 mm for spike and 2 cm for others. (**e**) Prediction of subcellular localization and cleavage site of ARE1 protein by TargetP 1.1 Server. Len, sequence length; cTP, chloroplast transit peptide; mTP, mitochondrial targeting peptide; SP, signal peptide of secretory pathway; Loc, prediction of localization; RC, reliability class; TPlen, predicted pre-sequence length; C, chloroplast. (**f**) Prediction of transmembrane helices in ARE1 protein by TMHMM 2.0 Server. The transmembrane, inside, and outside regions are indicated by red, blue and pink, respectively.


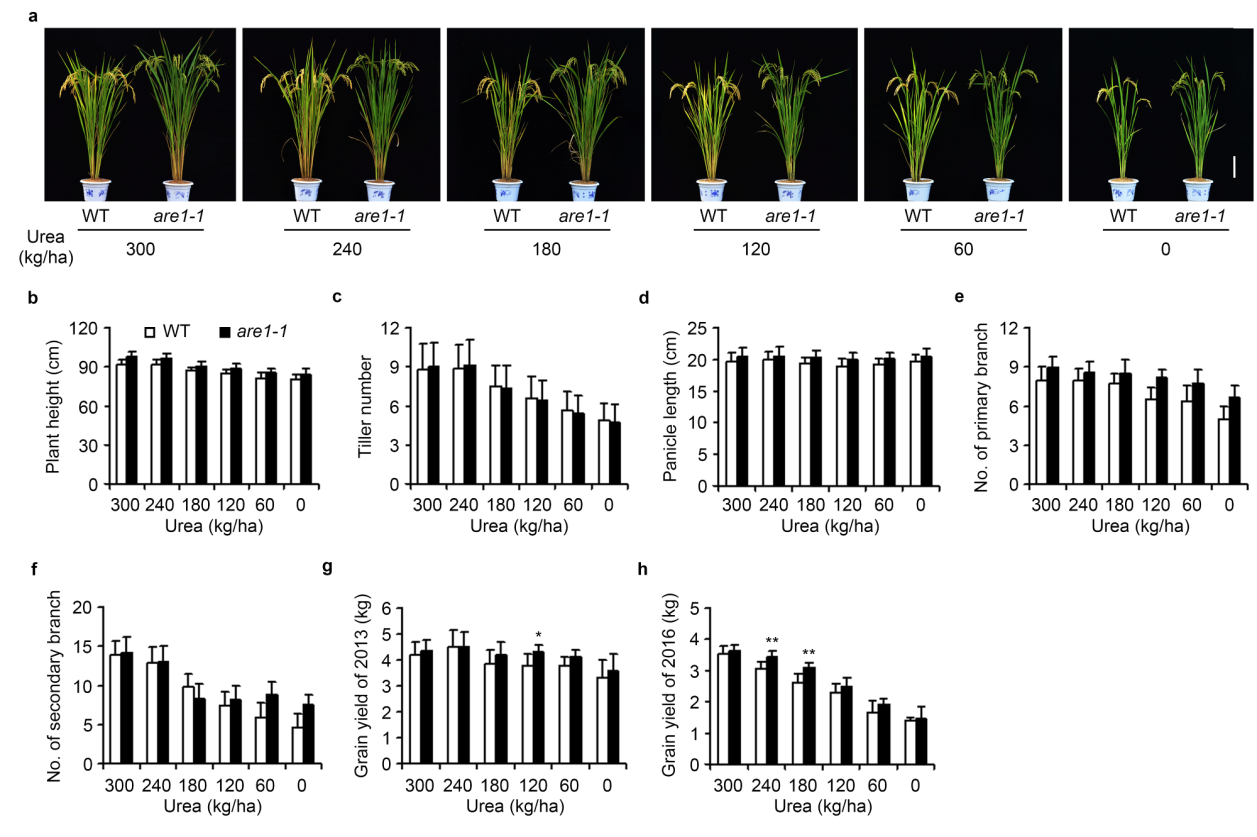


**Supplementary Figure 5 Enhanced grain yield by *are1*.** (**a**) Wild-type (WT; the *japonica* Nipponbare variety) and *are1-1* plants grown under various urea concentrations as indicated. Scale bar, 15 cm. (**b**-**f**) Quantitative analysis of the plant height, tiller number, panicle length, panicle primary branch number, and panicle secondary branch number of WT and *are1-1* plants grown under various urea concentrations as indicated (data collected in 2014). Data presented are mean values of 5 biological replicates with s.d. (**g**,**h**) Analysis of grain yield per plot of WT and *are1-1* plants grown under various urea concentrations as indicated in 2013 and 2016. Error bars in (**b**-**h**) indicate s.d. (*n* = 200 plants). * and ** indicate *P* < 0.05 and *P* < 0.01 (Student’s *t*-test), respectively.


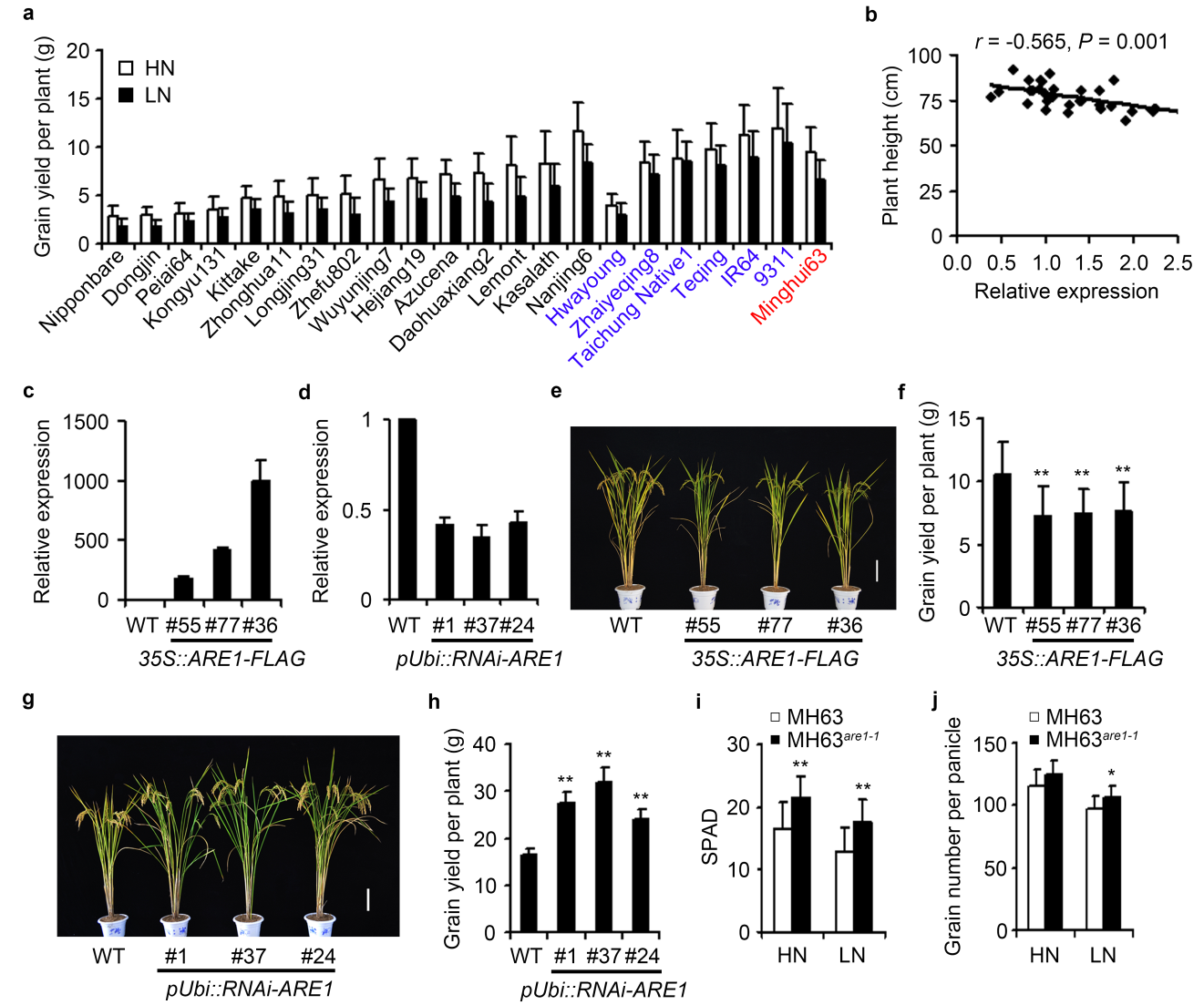


**Supplementary Figure 6 Modulation of grain yield by genetic variations in the *ARE1* promoter.** (**a**) Grain yield of various varieties grown under high nitrogen (HN; 300 kg/ha) or low nitrogen (LN; 150 kg/ha) conditions (*n* = 50 plants). Colored names of varieties indicate the genotype at the *ARE1* locus. See **Figure 5a**,**c** and **Figure 6a** for details. (**b**) Analysis of the correlation of plant height with the *ARE1* expression level in RILs shown in **Figure 6c**. *r* and *P* values are determined by the Pearson correlation analysis (*n* = 6 plants). (**c**,**d**) Analysis of *ARE1* expression in transgenic plants (in the Nipponbare background) carrying a 35S::*ARE1* (**c**) or an RNAi-*ARE1* transgene (**d**) by qRT-PCR. Data presented are mean values of 3 technical replicates with s.d. Numbers under the graph refer to the transgenic lines numbers. (**e**) 35S::*ARE1* transgenic plants at the dough stage. Scale bar, 15 cm. (**f**) Grain yield of the 35S::*ARE1* transgenic plants (*n* = 30 plants). (**g**) RNAi-*ARE1* transgenic plants at the dough stage. Scale bar, 15 cm. (**h**) Grain yield of the RNAi-*ARE1* transgenic plants grown under low nitrogen (180 kg/ha) conditions (*n* = 30 plants). (**i**,**j**) Analysis of soil-plant analysis development (SPAD) and grain yield of MH63 and MH63*^are1-1^* plants grown under high nitrogen (HN; 240 kg/ha) or low nitrogen (LN; 120 kg/ha) conditions (*n* = 25 plants). Error bars indicate s.d. * and ** indicate *P* < 0.05 and *P* < 0.01 (Student’s *t*-test), respectively.

**Supplementary Table 1 Analysis of total nitrogen contents**

| **Urea (kg/ha)** | **Organs** | **Total nitrogen content (mg/g DW^*^)** | |
| --- | --- | --- | --- |
|  |  | **Wild Type** | ***are1-1*** |
| 0 | Leaf blade | 11.96 ± 0.96^**^ | 12.92 ± 0.95 |
|  | Stem and sheath | 6.96 ± 1.14 | 6.81 ± 1.33 |
|  | Panicle | 14.97 ± 1.46 | 15.11 ± 1.56 |
|  | | | |
| 60 | Leaf blade | 11.17 ± 0.31 | 12.26 ± 0.81 |
|  | Stem and sheath | 6.37 ± 1.05 | 6.55 ± 1.06 |
|  | Panicle | 14.14 ± 1.20 | 15.62 ± 1.29 |
|  | | | |
| 120 | Leaf blade | 9.80 ± 0.68 | 11.60 ± 0.80 |
|  | Stem and sheath | 6.40 ± 0.79 | 6.80 ± 0.91 |
|  | Panicle | 14.05 ± 1.11 | 14.99 ± 1.28 |
|  | | | |
| 180 | Leaf blade | 9.93 ± 1.20 | 11.23 ± 1.70 |
|  | Stem and sheath | 6.74 ± 1.29 | 7.03 ± 1.19 |
|  | Panicle | 14.22 ± 1.21 | 15.75 ± 1.54 |
|  | | | |
| 240 | Leaf blade | 9.79 ± 0.53 | 10.43 ± 1.49 |
|  | Stem and sheath | 7.32 ± 1.09 | 7.69 ± 1.30 |
|  | Panicle | 15.07 ± 1.20 | 15.73 ± 1.84 |
|  | | | |
| 300 | Leaf blade | 10.26 ± 1.06 | 10.42 ± 1.22 |
|  | Stem and sheath | 7.39 ± 0.87 | 7.84 ± 1.23 |
|  | Panicle | 15.03 ± 1.19 | 15.94 ± 1.40 |

^*^DW: dry weight

^**^mean±s.d.

**Supplementary Table 2 Primers used in this study**

| Primer | Sequences (5'-3') | Experiments |
| --- | --- | --- |
| 35sF | CGACGGCCAGTGCCAAGCTTGCATG | p35S::ARE1-FLAG |
| 35sR | GCTGCAGGAGTCCCCCGTGTTCTCTCCAAATG | p35S::ARE1-FLAG |
| ARE1Gen1F | ATTGGCAAGGAATATGCCTCAACATGGGTC | pARE1::ARE1 |
| ARE1Gen1R | AGGAGTTTCTGGCTGCGCCTTACATCAAGC | pARE1::ARE1 |
| ARE1Gen2F | TGCAGAATTGATTACATGGAACATTACACA | pARE1::ARE1 |
| ARE1Gen2R | ATCGGCTTCAAGGCCGTAGTGCTCGAGAAT | pARE1::ARE1 |
| ARE1Gen3F | AAGAAGATTCGTCCAAAATGTTCAAAGAAGTT | pARE1::ARE1 |
| ARE1Gen3R | GAGCTCCATTCTTTCTTTTACTTAAATGTTAATTTG | pARE1::ARE1 |
| ARE1OeF | GCTGCAGATGAGCCGTTCCGCGGTAAG | p35S::ARE1-FLAG |
| ARE1OeR | ACCCGGGATGACGCTTTATTTCATCCAAAATG | p35S::ARE1-FLAG |
| ARE1ProF | CATCGTGGAAGGTTAGTTCCTACCC | pARE1::GUS |
| ARE1ProR | GCTGCAGGCTCATCAATCAAAGATCACGAAACG | pARE1::GUS |
| ARE1qrt5F | TAGCATTATTGATTGTGATTCCAT | qPCR |
| ARE1qrt5R | TGGAGGAGATTTACCAATCTCTACT | qPCR |
| ARE1RNAiF | CGGTACCACTAGTACACTGGTTCCCAAAGGGGTAG | pUbi::RNAi-ARE1 |
| ARE1RNAiR | CGGATCCGAGCTCCCTCCCACGAGCGCCCTTCCGC | pUbi::RNAi-ARE1 |
| ARE1Y47F | GAAGCTTCGCTTTTGCCAAGAAGAGGAGGCGGC | p35S::ARE1^Δ1-47^-YFP |
| ARE1YfpF | GAAGCTTCATGAGCCGTTCCGCGGTAAGCTCTG | p35S::ARE1-YFP |
| ARE1YfpR | GGTCGACTATGACGCTTTATTTCATCCAAAATG | p35S::ARE1/ARE1^Δ1-47^-YFP |
| GS1;2F | CTACTGTGGTATCGGTGCTGACAA | qPCR |
| GS1;2R | GTGATCCTCTCAAGAATGTAGCGA | qPCR |
| M34F | AACATCTGTCAGAAGAGGGCAGC | Genetic mapping |
| M34R | TATTGTTTTCACGGCTTCTTGTAGT | Genetic mapping |
| M36F | CCCCACACCACGTTGTTCTTT | Genetic mapping |
| M36R | CATTAAAAATAAGAAACACTTGGCAGTA | Genetic mapping |
| M79F | CGCACAAATAACGAAATGAGGATT | Genetic mapping |
| M79R | TACACAACAAACAAATGTAGTCTC | Genetic mapping |
| M81F | CGTCAACTGTTCGGGATAGAGGTATTAACG | Genetic mapping |
| M81R | TGCATTTCTTTAATAGGGTTTACTCTTGC | Genetic mapping |
| NADH-GOGAT1F | GCCAGGCGAGTATGCTGTCGGTAT | qPCR |
| NADH-GOGAT1R | AATCAGCCTCCGAACTTGAACTTT | qPCR |
| NosF | CGAGCTCGAATTTCCCCGATCGTTCAAAC | p35S::ARE1-FLAG |
| NosR | CGAATTCCCGATCTAGTAACATAGATGACACC | p35S::ARE1-FLAG |
| OsAMT1;1F | GGTCGTTCACCACCATCCTCAAGA | qPCR |
| OsAMT1;1R | GCAGACGTCGACCACGTTCCAGTG | qPCR |
| OsAMT1;2F | CCAACGGCTTCATCGGGAAGCACT | qPCR |
| OsAMT1;2R | GTGAGGAAGGCGGAGTAGATGAGG | qPCR |
| OsAMT1;3F | CATCACGTCCGGTTCCATCGCCGA | qPCR |
| OsAMT1;3R | GCGAAGTCGATGACGCCCGACTTG | qPCR |
| RM22613F | TTTCTGGCCCAGTTCAGTACAGC | Genetic mapping |
| RM22613R | TGGTGCGTACATATCCCTTTAACC | Genetic mapping |
| RM22633F | TTTCACCACTGTAGTCTCTCTCC | Genetic mapping |
| RM22633R | CTCGACAGTTTCTTAGCTAGTCC | Genetic mapping |
| RM22659F | GTCGTCGGAGACCACGATAGTCC | Genetic mapping |
| RM22659R | CGGCGCGCGACTACTATTACG | Genetic mapping |
| RM3374F | ACCGAGCAGACAAAGAGTAGC | Genetic mapping |
| RM3374R | TGGTGATCTAGGTCAAGTTGG | Genetic mapping |
| RM3481F | CCTCACGTCGTGCTCTCCAACC | Genetic mapping |
| RM3481R | CCTCGTCGCGTTCGTCAACC | Genetic mapping |
| RM3644F | TGCTCCTCCACCTACTACCATCC | Genetic mapping |
| RM3644R | GCAGAAATCTTGACAGAAGAGAGTGG | Genetic mapping |
| RM6838F | TCCTCCTCCACCTCAATCACACC | Genetic mapping |
| RM6838R | CCGAGCTCGCCATTAGCTTGC | Genetic mapping |
| RM8271F | AGCAGCTCCGATTGTGTTAGCC | Genetic mapping |
| RM8271R | AATGGCGTCTGTGGTACTTTGC | Genetic mapping |
| UBQ-F | ACCCTGGCTGACTACAACATC | qPCR |
| UBQ-R | AGTTGACAGCCCTAGGGTG | qPCR |
